# Association of Criminal Statutes for Opioid Use Disorder With Prevalence and Treatment Among Pregnant Women With Commercial Insurance in the United States

**DOI:** 10.1001/jamanetworkopen.2019.0338

**Published:** 2019-03-08

**Authors:** Laura E. Gressler, Savyasachi Shah, Fadia T. Shaya

**Affiliations:** 1 Pharmaceutical Health Services Research, University of Maryland School of Pharmacy, Baltimore

## Abstract

**Importance:**

Inadequate treatment of opioid use disorder (OUD) in pregnant women increases the risk of life-threatening consequences on maternal and fetal outcomes. Untreated OUD during pregnancy is associated with higher rates of adverse outcomes among newborns.

**Objective:**

To examine the variation in the prevalence of OUD and the use of medication-assisted treatment among commercially insured pregnant women according to region and state legislature.

**Design, Setting, and Participants:**

Cohort study in which the patient cohort used was derived from a 10% random sample of enrollees within the IQVIA PharMetrics Plus adjudicated claims and enrollment database from 2007 to 2015. The database consists of a 10% random sample of private health insurance recipients in the United States and contains claims and enrollment data that are representative of the commercially insured US population. The cohort comprised women (n = 110 285) between 18 and 45 years of age with a code indicating a delivery and continuous insurance enrollment 9 months before and 12 months after delivery. Data analysis was performed from December 2017 to May 2018.

**Exposures:**

Based on their state of residence, the women were classified into 4 different regions: South, Midwest, West, and Northeast. Those residing in states with statutes that imposed civil or criminal penalties for OUD diagnosis during pregnancy were placed in a separate population from those residing in states without these statutes.

**Main Outcomes and Measures:**

Diagnosis of OUD in the 9 months before delivery and the receipt of medication-assisted treatment in the 9 months before or 12 months after delivery.

**Results:**

The 110 285 pregnant women included in the analysis had a mean (SD) age of 30.26 (5.59) years, with most (67 771 [61.5%]) falling within the 26- to 35-year age range. Of this cohort, 277 women (0.25%) had a diagnosis of OUD and 312 (0.28%) received treatment. Among the 277 women with OUD, 127 (45.9%) received treatment. The prevalence of an OUD diagnosis and receipt of treatment within regions was statistically significant (OUD diagnosis by region: Midwest, 0.05%; North, 0.09%; South, 0.06%; West, 0.06%; χ^2^_3_ = 45.1148 [*P* < .001]; OUD treatment by region: Midwest, 0.05%; North, 0.08%; South, 0.10%; West, 0.05%; χ^2^_3_ = 26.5654 [*P* < .001]). The prevalence of OUD diagnosis was also statistically significant when comparing women residing in states with statutes with those in states without statutes (OUD diagnosis by criminal statutes: criminalization, 0.07%; no criminalization, 0.18%; χ^2^_1_ = 14.6456 [*P* < .001]; OUD treatment by criminal statutes: criminalization, 0.12%; no criminalization, 0.17%; χ^2^_1 _= 0.0895); the receipt of treatment was not statistically significant (*P* = .76).

**Conclusions and Relevance:**

These results appeared to show significant variations in the patterns of OUD diagnosis and receipt of medication-assisted treatment among pregnant women, suggesting the need to further explore the source of these variations.

## Key Points

**Question:**

Do the prevalence of opioid use disorder and the use of medication-assisted treatment among commercially insured pregnant women vary according to region and state legislature?

**Findings:**

In this cohort study of 110 285 pregnant women, statistically significant regional variations in opioid use disorder diagnosis and receipt of treatment were observed. Variations by state legislature were observed in opioid use disorder diagnoses but not in receipt of treatment.

**Meaning:**

These findings appear to indicate a discrepancy in the diagnosis and treatment of opioid use disorder on both a regional and legal basis.

## Teaser

This cohort study examines data from the IQVIA PharMetrics Plus database for prevalence of opioid use disorder and treatment among a random sample of privately insured pregnant women in 4 regions across the United States.

## Introduction

In the United States, opioid misuse is a growing and prevalent concern to the care and well-being of pregnant women as it can lead to adverse maternal and neonatal outcomes. Opioid use among women of childbearing age (15-44 years) has reached epidemic proportions, and a substantial increase in opioid use among pregnant women has been reported.^1,2^ Opioid use among pregnant women was approximately 5.6 per 1000 live births in 2012.^3,4^ In a previous study of the Optum commercially insured population, an opioid was dispensed to 14.4% (1 in every 7) of pregnant women and 2.2% were given an opioid 3 or more times during pregnancy, with some variation by region and state between 2005 and 2011.^5^ With the rising opioid use in this population comes a concurrent increase in opioid use disorder (OUD). *Opioid use disorder* is defined in the *Diagnostic and Statistical Manual of Mental Disorders *(Fifth Edition) as a problematic pattern of opioid use resulting in symptoms such as tolerance, withdrawal, craving, or an inability to cut down or control opioid use.^6,7^ Among all women hospitalized for delivery, opioid dependence increased from 0.17% in 1998 to 0.39% in 2011, representing a 127% increase.^8^

In pregnancy, opioid withdrawal is associated with decreased neonatal birth weight; illicit drug use; relapse; and resumption of high-risk behaviors such as intravenous drug use, prostitution, and criminal activity.^6,9^ Medication-assisted treatment, specifically methadone hydrochloride or buprenorphine hydrochloride, in combination with behavioral and psychosocial counseling is the standard practice for treating pregnant and postpartum women with OUD. Medication-assisted treatment minimizes opioid withdrawal, reduces risk-taking behavior, and decreases the risk of acquisition and transmission of infectious diseases.^10,11,12^ Various barriers exist for pregnant women to receive these treatments, such as accessibility, insurance coverage, region, and state laws that permit charges against pregnant women with OUDs. Such barriers are associated with a substantially decreased use of medication-assisted treatment.

Inadequate treatment of OUD in pregnant women is associated with increases in the risk of life-threatening consequences on maternal and fetal outcomes.^13,14,15^ Untreated OUD during pregnancy is associated with higher rates of low birth weight in the newborn, fetal growth restriction, and continued use of opioids by pregnant women.^16^ Exposure to opioids in utero places infants at risk for neonatal abstinence syndrome, a drug withdrawal syndrome that causes severe withdrawal symptoms, such as tremors, irritability, and respiratory distress, almost immediately after birth.^17^ The incidence of neonatal abstinence syndrome in newborns increased nationally from 3.4 to 5.8 per 1000 hospital births from 2009 to 2012.^3^ This spike represents an increased cost of $43 900 per hospital birth.^4^

Pregnant women with OUDs face not only medical consequences, such as an increased risk of obstetric morbidity and mortality, but also a predisposition to the potential loss of child custody and even criminalization in some states.^8,18^ A growing number of states over the past several years have debated and passed legislation criminalizing women with substance use disorders. Overall, 18 states since 2012 have required health professionals to report substance use disorder in pregnant women and have established civil or criminal laws that consider substance use to be child abuse.^19^

Escalating trends in OUD during pregnancy and the associated adverse maternal and neonatal outcomes have captured the attention of policymakers. The most recent initiatives are directed toward improving the accessibility and affordability of substance use treatment services, such as medication-assisted treatment for pregnant women.^20^ Most studies evaluating the prevalence of OUD and the use of medication-assisted treatment among pregnant women have either involved solely the Medicaid population or have shown that most women are covered by Medicaid.^4,8,19^ No studies thus far have shown the variations in prevalence of OUD and the use of medication-assisted treatment among pregnant women within a commercially insured population.

The objective of this study was therefore to examine the variation in the prevalence of OUD and the receipt of medication-assisted treatment among commercially insured pregnant women according to region and state legislature. We hypothesized that the diagnosis of OUD and receipt of treatment would vary by region and state legislature. Evaluating the prevalence of OUD and the receipt of medication-assisted treatment is important to define the current practices among commercially insured individuals and to inform future research within this population in the context of policy on civil or criminal laws on substance use.

## Methods

### Study Design and Setting

This retrospective observational cohort study included pregnant women with commercial insurance across the United States. Pregnant women were assessed for the outcomes 9 months before and 12 months after their recorded delivery date. The data obtained were deidentified, and the study was determined exempt from review by the University of Maryland Institutional Review Board. This study followed the Strengthening the Reporting of Observational Studies in Epidemiology (STROBE) reporting guideline.^21^

### Study Data

The patient cohort used in this study was derived from a 10% random sample of enrollees within the IQVIA PharMetrics Plus adjudicated claims and enrollment database from 2007 to 2015. The 10% random sample consisted of 12 416 600 enrollees. The IQVIA PharMetrics Plus database comprises fully adjudicated health plan claims data and enrollment information for commercially insured individuals.

The database receives information from health plans and self-insured employer groups throughout the United States for more than 150 million unique enrollees since 2006. This anonymous, patient-centric database includes all medical and pharmacy claims data (costs and descriptive services). Claims represent payments to medical practitioners for services rendered to individuals with health plan coverage. Procedural and diagnoses data are obtained from the medical claims file using *International Classification of Diseases, Ninth Revision, Clinical Modification* (*ICD-9-CM*) codes and *Current Procedural Terminology, Fourth Revision* (*CPT-4*) codes. The pharmacy claims information includes National Drug Codes, date of medication dispensed, quantity dispensed, and estimated days’ supply. The database also includes patient-level enrollment, which is a record of demographic variables, such as eligibility status (year of birth, sex, US census region, and eligibility by month). The enrollee population in the database is generally representative of individuals younger than 65 years and commercially insured, with a subset of individuals with commercial Medicare and Medicaid categorized by both age and sex. The mean length of enrollment is 39 or more months, and more than 30 million patients have 3 or more years of continuous enrollment (medical and pharmacy coverage). Each contributing plan’s data undergo a rigorous data quality review by IQVIA before their addition to the IQVIA PharMetrics Plus database.

### Identification of Sample

The study period spanned from June 30, 2007, to June 30, 2015. Female patients younger than 18 years and women older than 45 years were excluded from the cohort. To identify pregnancy, we collected 2995 *ICD-9-CM* codes and 88 *CPT-4* codes related to pregnancy. Codes were identified using published literature^22,23,24,25^ and the IQVIA PharMetrics Plus data dictionary. Based on the descriptions of the collected *ICD-9-CM* and *CPT-4* codes, the codes were classified into 1 of 8 categories by 3 researchers (including L.E.G. and S.S.) independently. Any disagreement in category classification of the code was discussed and resolved by the researchers. The 8 categories included pregnancy, complication of pregnancy, delivery, complication of delivery, miscarriage, complication of abortion, labor, and lactation. A code was classified as *delivery* if the description was indicative that a delivery occurred on the claim date. The date of the first claim indicating delivery was used as the index date. Women who only had a claim for a category other than delivery were excluded from the pregnancy cohort given that an index date could not be computed for them. Women were required to be continuously enrolled 9 months before the index date and 12 months after the index date to identify the outcomes.

The primary outcome was a diagnosis of OUD during the 9 months before the index date.^26^ From the published literature, we used 20 *ICD-9-CM* codes to identify an OUD.^27^

Receipt of medication-assisted treatment was observed 9 months before the index date to reflect the mean duration of pregnancy and 12 months after the index date. The postdelivery time frame of 12 months was chosen to allow ample time after delivery to capture receipt of treatment. This time frame was primarily a concern for women who lived in states with statutes criminalizing OUD. Receipt of treatment was recorded if National Drug Codes for methadone, buprenorphine, or naltrexone hydrochloride were present during this duration.

On the basis of state of residence, women were classified into 4 different regions: South, Midwest, West, and Northeast and further categorized into 2 populations.^28^ The first population resided in any of the 18 states with statutes that imposed civil or criminal penalty on women with OUD during pregnancy. The 18 states were as follows: Arkansas, Colorado, Florida, Illinois, Indiana, Iowa, Louisiana, Minnesota, Nevada, Oklahoma, Rhode Island, South Carolina, South Dakota, Texas, Virginia, Wisconsin, Alabama, and Tennessee.^19^ The second population resided in states without these statutes.

### Statistical Analysis

Data analysis was performed from December 2017 to May 2018. The prevalence of OUD diagnosis in the commercially insured US population was calculated using the unique number of pregnant women between 18 and 45 years of age with an OUD diagnosis during the 8-year study period as the numerator and the total number of pregnant women within the age restriction as the denominator. The prevalence of treatment was calculated in a similar manner. Prevalence calculations were stratified by region and by the presence of legislation that criminally or civilly prosecutes pregnant women with an OUD diagnosis. All states categorized as having such legislation had passed laws before 2012 and had cases convicting women before 2012.^19^ Cochran-Mantel-Haenszel (χ^2^) statistics were calculated to determine whether a substantial difference exists between the prevalence of OUD diagnoses and receipt of treatment by region and by presence of civil or criminal statutes. Statistical significance was determined at 2-sided *P* = .05 using a 2-sided Pearson χ^2^ test. All statistical analyses were performed in SAS, version 9.4 (SAS Institute Inc).

## Results

**Figure 1.**
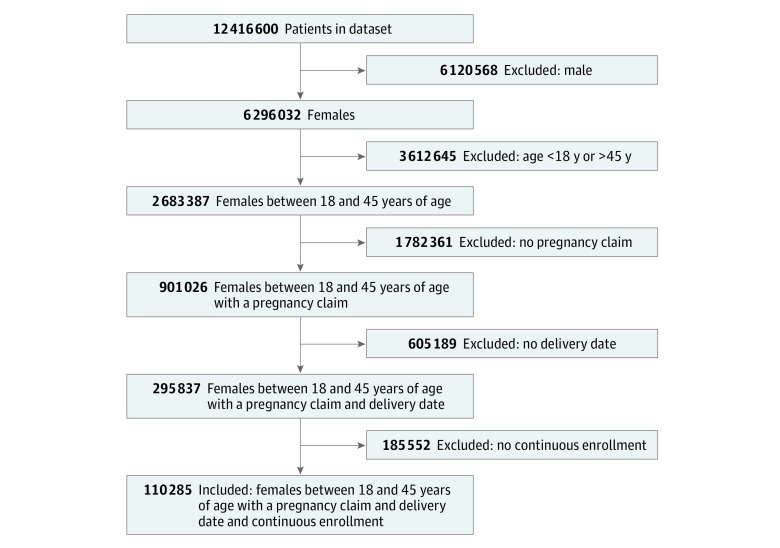
Inclusion and Exclusion Criteria

Of a total 12 416 600 individuals in the commercially insured population, 2 683 387 (21.6%) were women between 18 and 45 years of age, of whom 295 837 (11.0%) had a code for a delivery date. The final cohort consisted of 110 285 pregnant women between 18 and 45 years of age who were continuously enrolled 9 months before the delivery date and 12 months after the delivery date (Figure 1). The women had a mean (SD) age of 30.26 (5.59) years, with most (67 771 [61.5%]) falling within the 26- to 35-year age range. The women were primarily commercially insured (68 356 [62.0%]) or self-insured or other/unknown (34 361 [31.2%]). Medicaid was the primary payer for 7568 pregnant women (6.9%) in the sample. Some categories are collapsed owing to the small sample sizes that could not be reported. Demographic data are summarized in Table 1.

**Table 1.** Demographics of the Sample

Variable	No. (%)		
	All Women	Women With an Opioid Use Disorder Diagnosis	Women Receiving Medication-Assisted Treatment
Total	110 285	277	312
Age, mean (SD), y	30.26 (5.59)	28.54 (6.37)	29.97 (6.68)
Age category, y			
18-25	17 545 (15.9)	94 (33.9)	81 (25.9)
26-35	67 771 (61.5)	133 (48.0)	157 (50.3)
36-45	24 969 (22.6)	50 (18.1)	74 (23.7)
Insurance type			
Commercial	68 356 (62.0)	135 (48.7)	171 (54.8)
Medicaid	7568 (6.9)	70 (25.3)	51 (16.4)
Self-insured or other/unknown	34 361 (31.2)	72 (25.9)	90 (28.8)
Region			
Midwest	32 228 (29.2)	52 (18.8)	52 (16.7)
Northeast	23 066 (20.9)	98 (35.4)	87 (27.9)
South	34 621 (31.4)	66 (23.8)	113 (36.2)
West	20 370 (18.5)	61 (22.0)	60 (19.2)
Legislation present			
Yes	44 683 (40.5)	81 (29.2)	129 (41.4)
No	65 602 (59.5)	196 (70.8)	183 (58.7)
Received treatment	312 (0.28)	127 (45.9)	312 (100)
Treatment type			
Buprenorphine hydrochloride	215 (0.19)	105 (37.9)	215 (68.9)
Methadone/naltrexone hydrochloride	97 (0.09)	22 (7.9)	97 (31.1)

Among the 110 285 pregnant women, 277 (0.25%) had a recorded diagnosis of OUD and 312 (0.28%) received medication-assisted treatment. Women with a recorded diagnosis of OUD had a mean (SD) age of 28.54 (6.37) years and were primarily insured commercially (135 [48.7%]) or by Medicaid (70 [25.3%]). Buprenorphine was the primary medication dispensed among women with an OUD diagnosis. One hundred five women (37.9%) received buprenorphine, and 22 (7.9%) received methadone or naltrexone. Among 312 women who received treatment, the mean (SD) age was 29.97 (6.68) years. These women were primarily covered by commercial insurance (171 [54.8%]) or were self-insured or other/unknown (90 [28.8%]). Among the treated pregnant women, 215 (68.9%) received buprenorphine and 97 (31.1%) received methadone or naltrexone.

In the Midwest, 52 women (0.05%) had an OUD diagnosis and 52 (0.05%) received treatment, and these numbers represented the lowest prevalence of OUD diagnoses and receipt of treatment among all regions. In contrast, the Northeast had a higher prevalence of OUD diagnosis, representing 98 women (0.09%), and a higher prevalence of treatment, representing 87 women (0.08%). The South also had a higher rate of women receiving treatment, representing 113 women [0.10%], but had a lower prevalence of OUD diagnoses, representing 66 women (0.06%), which indicates that more women received treatment than received a diagnosis. In the West, 61 women (0.06%) had an OUD diagnosis and 60 women (0.05%) received treatment. The differences in prevalence of OUD diagnoses and receipt of treatment within regions were statistically significant (OUD diagnosis by region: Midwest, 0.05%; North, 0.09%; South, 0.06%; West, 0.06%; χ^2^_3_ = 45.1148 [*P* < .001]; OUD treatment by region: Midwest, 0.05%; North, 0.08%; South, 0.10%; West, 0.05%; χ^2^_3_ = 26.5654 [*P* < .001]) (Table 2).

**Table 2.** Prevalence of Opioid Use Disorder Diagnosis and Treatment of Pregnant Women by Region and Presence of Legislation

Variable	All Women	Women With an Opioid Use Disorder Diagnosis, No. (%)	*P* Value	Women Receiving Medication-Assisted Treatment, No. (%)	*P* Value
Region					
Midwest	32 228	52 (0.05)	<.01	52 (0.05)	<.01
Northeast	23 066	98 (0.09)		87 (0.08)	
South	34 621	66 (0.06)		113 (0.10)	
West	20 370	61 (0.06)		60 (0.05)	
Legislation present					
Yes	44 683	81 (0.07)	<.01	129 (0.12)	.76
No	65 602	196 (0.18)		183 (0.17)	
All	110 285	277 (0.25)		312 (0.28)	

**Figure 2.**
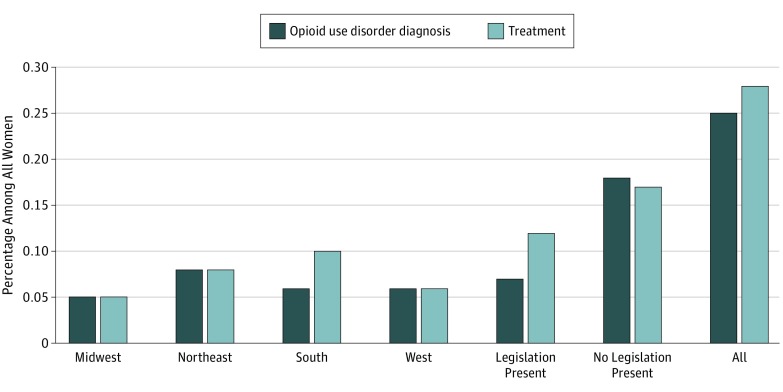
Prevalence of Opioid Use Disorder Diagnosis and Treatment

Of the 44 683 women living in states in which women with OUD diagnoses were civilly or criminally prosecuted, 81 (0.07%) had a diagnosis code for OUD and 129 (0.12%) received treatment. The prevalence of diagnosed OUD and the prevalence of treatment were higher in states without these statutes. Of the 65 602 women living in states with no substance use criminalization statutes, 196 (0.18%) had an OUD diagnosis and 183 (0.17%) received treatment (Table 2). The prevalence of OUD diagnoses was statistically significant (OUD diagnosis by criminal statutes: criminalization, 0.07%; no criminalization, 0.18%; χ^2^_1_ = 14.6456 [*P* < .001]; OUD treatment by criminal statutes: criminalization, 0.12%; no criminalization, 0.17%; χ^2^_1 _= 0.0895), but the prevalence of treatment receipt was not statistically significant (*P* = .76) (Table 2). All prevalence rates are illustrated in Figure 2. In summary, in a cohort of 110 285 commercially insured pregnant women, 25 in every 10 000 women had a recorded OUD diagnosis and 28 in every 10 000 received treatment. Variations by region and by presence of criminal or civil statutes were observed in the diagnosis of OUD. Statistically significant regional variations were observed in the receipt of treatment. No statistically significant variations were observed in the receipt of treatment by presence of criminal or civil statutes. Of the 277 women with an OUD diagnosis, only 127 (45.9%) received medication-assisted treatment.

## Discussion

These results differ from findings in a similar study by Angelotta et al,^19^ who found that criminal or civil statutes did affect whether a pregnant woman would receive treatment. Note that the population in the Angelotta et al^19^ study included a Medicaid population, in which OUD was more prevalent and treatment more likely to be covered than in commercially insured populations, such as this study population. Medicaid patients are twice as likely as patients with no insurance or private insurance to receive OUD treatment.^29^ Bateman et al^5^ explored the Optum data and found results similar to those in the present study, showing a low prevalence of methadone and buprenorphine dispensing for commercially insured pregnant women. Bateman et al^5^ found that buprenorphine was dispensed to 0.03% of women during pregnancy and methadone was dispensed to 0.02% of women in pregnancy. Among the women exposed to opioids during pregnancy, 0.5% had a diagnosis of opioid dependence and 0.02% had an OUD diagnosis.^5^

A notable finding in the present study pertains to the analysis by region and by civil and criminal statutes. Regional variations may account for the limited availability of treatment centers or practitioners specializing in medication-assisted treatment within a region. This lack would indicate that even if practitioners who treat the commercially insured have good practices and want to provide treatment, they may not be able to send women to receive care. We found a lower prevalence of recorded OUD and lower prevalence of medication-assisted treatment in states with statutes civilly or criminally prosecuting women with an OUD diagnosis.

These results show a statistically significant association between the presence of these statutes and a lower prevalence of recorded OUD. The low prevalence of OUD in the states with statutes may be the result of fewer women seeking care for OUD, perhaps out of the fear of being criminally charged and losing custody of their newborn. In addition, the same fear compounded by stigma may lead some women to avoid receiving care from medical practitioners, even when they have insurance coverage. One study that interviewed pregnant women who were substance users reported that 73% feared being identified as substance users, and many often used tactics, such as skipping appointments or forgoing prenatal care altogether, to avoid any detection of their drug use.^15^

### Limitations and Strengths

Even as we note the importance of these findings, we also advise that they should be carefully considered because of the inherent limitations of claims data. First, practitioners may be hesitant to code for an OUD diagnosis for a number of reasons, including the concern that the patient may face consequences and may no longer seek prenatal care. Second, information in commercial claims databases is limited. The database does not contain information on the gestational age at birth. Given that women with OUD are more likely to give birth preterm, the look-back period of 9 months from delivery may yield a conservative estimate and may be longer than some pregnancies.^8^ Furthermore, women may have received medication-assisted treatment from a separate facility specializing in substance use disorder, such as a freestanding methadone clinic. These facilities may not bill the insurance, and therefore treatment would not be captured in the database. Third, women may be less likely to seek care for their OUD and may not report the diagnosis or treatment to their insurance because the plan does not cover treatment. Even if a claim is entered, pharmacy claims measure whether the medication was dispensed and do not indicate whether the medication was actually taken by the patient. Moreover, pharmacy claims do not capture medications dispensed to patients during hospitalizations. A number of women with an OUD diagnosis may have been hospitalized and received treatment during an inpatient stay. This inpatient treatment would not have been captured in our analysis. All of these limitations may underestimate the prevalence of OUD and medication-assisted treatment. The results of this study are not generalizable to women covered by other forms of insurance.

However, despite the challenges of identifying pregnancy and treatment frequency in claims databases, several strengths of this study should be noted. This study is the first, to our knowledge, to attempt to identify the OUD prevalence and treatment during pregnancy within the IQVIA PharMetrics Plus database commercially insured population. We used a novel method of identifying pregnancy within claims and found a possible underdiagnosis and treatment of OUD among pregnant women.

The most striking finding and contribution from this study to an ongoing effort to address the opioid epidemic is that the criminality statute is the basis of geographic variations. The process leading to the unintended consequences of such statutes is very complex and should be examined very carefully lest it produces the opposite of its intended purpose. The geographic variations indicate that differential factors within regions may play a role in the diagnosis of OUD among pregnant women.

## Conclusions

The prevalence of OUD in commercially insured pregnant women and receipt of treatment within the United States appeared to vary by region. The diagnosis of OUD also significantly varied by the presence of criminal or civil statutes within the state of residence, whereas the receipt of treatment did not. The proportion of women with OUD and receiving treatment within this insured population was relatively low compared with women in other populations. These findings indicate a discrepancy in the diagnosis and treatment of OUD both on a regional and a legal basis. This study demonstrates the need for further studies into policies that reduce stigma and discrimination and that encourage the proper identification of OUD during pregnancy as part of a national quest to address the opioid epidemic among society’s most vulnerable women.

## References

[1] Krans EE, Patrick SW Opioid use disorder in pregnancy: health policy and practice in the midst of an epidemic. Obstet Gynecol. 2016;128(1):-. doi:10.1097/AOG.0000000000001446 27275812 PMC4917415

[2] Jarlenski M, Barry CL, Gollust S, Graves AJ, Kennedy-Hendricks A, Kozhimannil K Polysubstance use among US women of reproductive age who use opioids for nonmedical reasons. Am J Public Health. 2017;107(8):1308-1310. doi:10.2105/AJPH.2017.303825 28640680 PMC5508143

[3] Patrick SW, Davis MM, Lehman CU, Cooper WO Increasing incidence and geographic distribution of neonatal abstinence syndrome: United States 2009 to 2012. J Perinatol. 2015;35(8):667. doi:10.1038/jp.2015.63 26219703

[4] Patrick SW, Schumacher RE, Benneyworth BD, Krans EE, McAllister JM, Davis MM Neonatal abstinence syndrome and associated health care expenditures: United States, 2000-2009. JAMA. 2012;307(18):1934-1940. doi:10.1001/jama.2012.3951 22546608

[5] Bateman BT, Hernandez-Diaz S, Rathmell JP, Patterns of opioid utilization in pregnancy in a large cohort of commercial insurance beneficiaries in the United States. Anesthesiology. 2014;120(5):1216-1224. doi:10.1097/ALN.0000000000000172 24525628 PMC3999216

[6] Dashe JS, Jackson GL, Olscher DA, Zane EH, Wendel GD Jr Opioid detoxification in pregnancy. Obstet Gynecol. 1998;92(5):854-858. 9794682 10.1016/s0029-7844(98)00312-3

[7] American Psychiatric Association Diagnostic and Statistical Manual of Mental Disorders. 5th ed. Arlington, VA: American Psychiatric Association; 2013.

[8] Maeda A, Bateman BT, Clancy CR, Creanga AA, Leffert LR Opioid abuse and dependence during pregnancy: temporal trends and obstetrical outcomes. Anesthesiology. 2014;121(6):1158-1165. doi:10.1097/ALN.0000000000000472 25405293

[9] Stewart RD, Nelson DB, Adhikari EH, The obstetrical and neonatal impact of maternal opioid detoxification in pregnancy. Am J Obstet Gynecol. 2013;209(3):267.e1-267.e5. doi:10.1016/j.ajog.2013.05.026 23727040

[10] Fullerton CA, Kim M, Thomas CP, Medication-assisted treatment with methadone: assessing the evidence. Psychiatr Serv. 2014;65(2):146-157. doi:10.1176/appi.ps.201300235 24248468

[11] Thomas CP, Fullerton CA, Kim M, Medication-assisted treatment with buprenorphine: assessing the evidence. Psychiatr Serv. 2014;65(2):158-170. doi:10.1176/appi.ps.201300256 24247147

[12] Jones HE, Deppen K, Hudak ML, Clinical care for opioid-using pregnant and postpartum women: the role of obstetric providers. Am J Obstet Gynecol. 2014;210(4):302-310. doi:10.1016/j.ajog.2013.10.010 24120973 PMC7213596

[13] Hand DJ, Short VL, Abatemarco DJ Substance use, treatment, and demographic characteristics of pregnant women entering treatment for opioid use disorder differ by United States census region. J Subst Abuse Treat. 2017;76:58-63. doi:10.1016/j.jsat.2017.01.011 28161143

[14] Patrick SW, Buntin MB, Martin PR, Barriers to accessing treatment for pregnant women with opioid use disorder in Appalachian states [published online October 9, 2018]. Subst Abus. doi:10.1080/08897077.2018.1488336 PMC9069995 29949454

[15] Stone R Pregnant women and substance use: fear, stigma, and barriers to care. Health Justice. 2015;3:2. doi:10.1186/s40352-015-0015-5

[16] Klaman SL, Isaacs K, Leopold A, Treating women who are pregnant and parenting for opioid use disorder and the concurrent care of their infants and children: literature review to support national guidance. J Addict Med. 2017;11(3):178-190. doi:10.1097/ADM.0000000000000308 28406856 PMC5457836

[17] Kaltenbach K, Berghella V, Finnegan L Opioid dependence during pregnancy: effects and management. Obstet Gynecol Clin North Am. 1998;25(1):139-151. doi:10.1016/S0889-8545(05)70362-4 9547764

[18] National Institute on Drug Abuse How does heroin use affect pregnant women? https://www.drugabuse.gov/publications/research-reports/heroin/how-does-heroin-abuse-affect-pregnant-women. Updated June 2018. Accessed February 18, 2018.

[19] Angelotta C, Weiss CJ, Angelotta JW, Friedman RA A moral or medical problem? the relationship between legal penalties and treatment practices for opioid use disorders in pregnant women. Womens Health Issues. 2016;26(6):595-601. doi:10.1016/j.whi.2016.09.002 27773527

[20] Kremer ME, Arora KS Clinical, ethical, and legal considerations in pregnant women with opioid abuse. Obstet Gynecol. 2015;126(3):474-478. doi:10.1097/AOG.0000000000000991 26244538

[21] von Elm E, Altman DG, Egger M, Pocock SJ, Gøtzsche PC, Vandenbroucke JP; STROBE Initiative The Strengthening the Reporting of Observational Studies in Epidemiology (STROBE) statement: guidelines for reporting observational studies. J Clin Epidemiol. 2008;61(4):344-349. doi:10.1016/j.jclinepi.2007.11.008 18313558

[22] Li Q, Andrade SE, Cooper WO, Validation of an algorithm to estimate gestational age in electronic health plan databases. Pharmacoepidemiol Drug Saf. 2013;22(5):524-532. doi:10.1002/pds.3407 23335117 PMC3644383

[23] Shakib JH, Korgenski K, Sheng X, Varner MW, Pavia AT, Byington CL Tetanus, diphtheria, acellular pertussis vaccine during pregnancy: pregnancy and infant health outcomes. J Pediatr. 2013;163(5):1422-6.e1-4. doi:10.1016/j.jpeds.2013.06.021 23896191 PMC4102585

[24] Hornbrook MC, Whitlock EP, Berg CJ, Development of an algorithm to identify pregnancy episodes in an integrated health care delivery system. Health Serv Res. 2007;42(2):908-927. doi:10.1111/j.1475-6773.2006.00635.x 17362224 PMC1955367

[25] Ailes EC, Simeone RM, Dawson AL, Petersen EE, Gilboa SM Using insurance claims data to identify and estimate critical periods in pregnancy: an application to antidepressants. Birth Defects Res A Clin Mol Teratol. 2016;106(11):927-934. doi:10.1002/bdra.23573 27891779 PMC5225464

[26] Knox CA, Delaney JA, Winterstein AG Anti-diabetic drug utilization of pregnant diabetic women in US managed care. BMC Pregnancy Childbirth. 2014;14:28. doi:10.1186/1471-2393-14-28 24438493 PMC3898248

[27] Gressler LE, Martin BC, Hudson TJ, Painter JT Relationship between concomitant benzodiazepine-opioid use and adverse outcomes among US veterans. Pain. 2018;159(3):451-459. doi:10.1097/j.pain.0000000000001111 29189516

[28] US Census Bureau Census regions and divisions of the United States. https://www2.census.gov/geo/pdfs/maps-data/maps/reference/us_regdiv.pdf. Accessed November 2018.

[29] Kaiser Family Foundation The opioid epidemic and Medicaid's role in facilitating access to treatment. https://www.kff.org/medicaid/issue-brief/the-opioid-epidemic-and-medicaids-role-in-facilitating-access-to-treatment/. Updated 2018. Accessed September 4, 2018.

